# Mental health progress requires causal diagnostic nosology and scalable causal discovery

**DOI:** 10.3389/fpsyt.2022.898789

**Published:** 2022-11-15

**Authors:** Glenn N. Saxe, Leonard Bickman, Sisi Ma, Constantin Aliferis

**Affiliations:** ^1^Department of Child and Adolescent Psychiatry, New York University Grossman School of Medicine, New York, NY, United States; ^2^Ontrak Health, Inc., Henderson, NV, United States; ^3^Department of Psychology, Florida International University, Miami, FL, United States; ^4^Program in Data Science, Department of Medicine, Clinical and Translational Science Institute, Institute for Health Informatics, School of Medicine, University of Minnesota, Minneapolis, MN, United States

**Keywords:** psychiatry, mental health, causality, etiology, diagnosis, methodology, outcomes

## Abstract

Nine hundred and seventy million individuals across the globe are estimated to carry the burden of a mental disorder. Limited progress has been achieved in alleviating this burden over decades of effort, compared to progress achieved for many other medical disorders. Progress on outcome improvement for all medical disorders, including mental disorders, requires research capable of discovering causality at sufficient scale and speed, and a diagnostic nosology capable of encoding the causal knowledge that is discovered. Accordingly, the field’s guiding paradigm limits progress by maintaining: (a) a diagnostic nosology (DSM-5) with a profound lack of causality; (b) a misalignment between mental health etiologic research and nosology; (c) an over-reliance on clinical trials beyond their capabilities; and (d) a limited adoption of newer methods capable of discovering the complex etiology of mental disorders. We detail feasible directions forward, to achieve greater levels of progress on improving outcomes for mental disorders, by: (a) the discovery of knowledge on the complex etiology of mental disorders with application of Causal Data Science methods; and (b) the encoding of the etiological knowledge that is discovered within a causal diagnostic system for mental disorders.

## Introduction

The limited progress achieved in reducing the burden of mental disorders, over decades, is of growing concern (1–4). Recently, Leichsenring et al. conducted an umbrella meta-analysis on the intervention effects from 102 meta-analyses on 3,782 Randomize Clinical Trials (RCTs) for 650,514 participants concerning most major mental disorders. They concluded that the effects of most interventions for most disorders were small, and that bias in reporting of results was commonly observed (5).

At present, 970 million individuals across the globe are estimated to carry the burden of a mental disorder and await intervention progress (6). By comparison, extraordinary progress has been achieved in preventing and treating many other medical disorders, such as heart disease, cancer, AIDS, COVID-19 and other infectious diseases, stroke, and diabetes (7–12).

The present article is not the first to highlight the importance of causal knowledge for understanding the nature of mental disorders (13–16), but attempts a detailed analysis as to why causal knowledge is necessary for achieving progress in lessening their burdens, informed by innovative data science methods. A fundamental premise of our analysis is that outcomes for mental disorders—like for all medical disorders—cannot improve without discovery of causal knowledge governing these outcomes (17, 18). As will be shown, there is a structurally driven dearth of scientific causal knowledge on mental disorders, because the research methods conventionally employed to study etiology cannot infer causation at sufficient scale and speed. Moreover, the encoding of etiological knowledge for diagnosis (that invariably guides treatments) is precluded by the form of diagnostic nosology conventionally practiced by the field for mental disorders. These central components to the field’s guiding scientific paradigm serve to impose great limitations to progress for improving outcomes for mental disorders.

In the present work, we identify the wide net of interwoven effects of lack of causality in research and practice for mental disorders that structurally and systematically create such a barrier to progress. Then, we outline how the paradigm can be shifted to yield the causal knowledge necessary for this barrier to be broken.

We start by defining causation in relation to improving outcomes for all medical disorders.

## The problem with the paradigm: Scientific methods Ill-suited to discover causes and diagnostic nosology incapable of encoding causal knowledge

### How advances in outcome improvement for medical disorders are achieved: The causal-exclusivity thesis

The pursuit of advances in any health science requires the capacity to change the probability of health outcomes through interventions, and the reliable and accurate prediction of outcome events given such interventions. The latter may be preventative or therapeutic actions at the individual or population levels. Interventions lead to changes in the probability of outcomes that are within the control of scientists, policy makers, or clinicians.

A causal factor is a measurable variable that—when an intervention changes its value—results in an alteration in the probability of a future outcome event compared to the probability of the outcome *in the absence of interventions* on *that factor*. Conversely, interventional changes in the value of a factor that is non-causal of a future event, cannot lead to changes in the probability of that future outcome, even if those non-causal factors’ values are strongly predictive of the outcome independent of the interventions on that factor (17–22).

These premises on the nature of causation entail a general set of consequences that apply to every health field. We state them in the form of a central thesis, *The Causal-Exclusivity Thesis (CET)*:

*Outcomes can be systematically improved through intervention on their causes, and in no other way. Therefore, a health field without the tools to distinguish causes from non-causal correlates – and without a diagnostic nosology to encode this distinction* – *cannot advance.*^1^

The discussion of the ramifications of the CET to the mental health field will become clearer by examining the history of progress for one mental disorder in particular, Major Depression (MD), in comparison to another common and debilitating disorder, Myocardial Infarction (MI). This comparison, which can be readily generalized across mental disorders vs non-mental disorders, reveals the lack of progress in the study of the causes, consequences, and persistence of disorders in the mental health field. As we will show, this comparison is informative regardless of one’s belief in the *differences in levels of complexity* among these disorders or in the *differences in the challenge to discovering their causes*, because a diagnostic nosology can contribute to outcome improvement only by being informed on causes.

### The application of diagnostic nosology to improve outcomes: A comparison of major depression and myocardial infarction since 1980

In the late 1970s, several theories on the etiology of MD were prominent. Biologically-oriented mental health specialists embraced etiological theory related to deficiencies in synaptic transmission of norepinephrine and serotonin (23–25). Psychoanalytically-oriented specialists embraced etiological theory related to unconscious self-blame/anger for interpersonal losses (26, 27). Cognitive behaviorally oriented specialists embraced etiological theory related to negative beliefs about the self, the world, and the future (28, 29). The American Psychiatric Association (APA) decided to upgrade its diagnostic system from DSM II (30), in the late 1970s but the field could not reach a consensus on the etiology of MD (or on the etiology of other psychiatric disorders). With the release of DSM III in 1980, the APA (31) decided to define psychiatric disorders as specific patterns of objectively observable symptoms without regard to causation.

This rationale was stated in an influential editorial by the former director of the federal *Alcohol, Drug Abuse and Mental Health Administration* shortly after DSM III’s release: “… *reliance on descriptive rather than etiologic criteria does not represent an abandonment of the ideal of modern scientific medicine that classification and diagnosis should be by causation. Rather, it represents a strategic mode of dealing with the frustrating reality that, for most of the disorders we currently treat, there is only limited evidence for their etiologies.*… *for most disorders the evidence is insufficient and inconclusive”* (32).

In other words, because of the lack of causal knowledge of mental disorders, it was decided, as a strategy, that causation would be expunged from the nosology, leading to, as will be described, tragic consequences. That DSM-5 was released in 2013, still with a recurrent non-causal nosology, is a strong acknowledgment of the lack of progress on causal knowledge of mental disorders in the decades after DSM III was released.

Table 1 summarizes the historic development of MD criteria (as a representative example of a nexus of translational research and related criteria in psychiatry), in stark contrast to MI criteria (as a representative example of corresponding translational criteria and research outside psychiatry), from the 1980s to the present time.

**TABLE 1:** Historical evolution of diagnostic criteria and underlying research for MD and MI as representative examples of psychiatric and non-psychiatric research and practice.

**Major Depression**
In 1980: when DSM III was released, a depressive episode was defined as a two-week period of having persistently low mood and/or loss of interest in activities that typically provide pleasure (criterion A) and having at least four out of eight additional symptoms including insomnia, loss of appetite, feelings of worthlessness, hopelessness helplessness and suicidal thoughts (criterion B). A diagnosis of MD was made if the individual met criteria for an episode of depression, and it led to functional impairment, and several exclusionary criteria were not met such as organic etiology, symptoms of mania or psychosis, or grief reactions (31). 1994: DSM IV adjusted the criteria by eliminating the criterion A and B distinction and offering nine symptom groups (including persistently low mood and loss of interest—formerly criterion A) and requiring the meeting at least five of nine symptoms, as long as both low mood and loss of interest were included. DSM IV added the distinction between a single depressive episode and recurrent depressive episodes (more than one depressive episodes) (121). 2013: DSM-5, adjusted the criteria in minor ways. Almost identical nine symptom categories were maintained from DSM IV for which five of nine must be endorsed. Changes included the addition of a “with mixed features” qualifier, if up to three manic symptoms were present. The bereavement exclusion was also removed as it was believed that MD can follow bereavement reactions (35).
**Myocardial Infarction**
Early 20th century: The origins of this understanding can be traced as described in the excellent historical account of MI in: http://www.epi.umn.edu/cvdepi/essay/history-of-heart-attack-diagnosis-and-understanding/. Whereas various syndromes describing or overlapping with the modern concept of MI were known from antiquity, it was not until the invention of electrocardiography and the early 20th century studies linking MI to coronary atherosclerosis, that this disease was properly understood. Researchers at the time pursued and causally linked EKG patterns with alterations of cardiac muscle conductivity and these with reduced blood supply due to coronary artery stenosis. 1980: Clear consensus in the field of Cardiology that the etiology of MI was fundamentally related to ischemic damage to cardiac tissue. The set of clinical symptoms/signs—exertional chest pain, shortness of breath, EKG changes (q waves, ST elevations) and elevation of cardiac enzymes (e.g., Myoglobin, Total CK, CK-MB)—comprised the diagnostic criteria for MI. In 1980, there was emerging consensus that CK-MB was the cardiac enzyme with highest specificity for cardiac tissue necrosis and its use was becoming part of the standards of practice (122). 2000: The diagnosis of MI was redefined and the reliance on CK-MB was changed to serial measurement with cardiac troponins, as troponins were found to be much more sensitive and specific for detecting damage to cardiac tissue (123). Present time: MI is classified based on the presence or absence of ST segment elevation on EKG (ST Elevation MI/STEMI or Non-ST Elevation MI/NSTEMI) as STEMI indicates full coronary artery occlusion whereas NSTEMI does not. Diagnoses of Myocardial Infarction is then further classified into six categories, all based on etiology. Sequential assessment of troponins continues to be an essential component of the diagnosis of Myocardial Infarction (124, 125).

The very substantial improvements in morbidity and mortality in MI prognosis since 1980 are significantly related to such improvements in diagnosis and etiology-driven treatments targeting disorder mechanisms (33, 34). In contrast, decisions on diagnostic criteria for MD—and their refinements—were not clearly tied to evidence on prognostic prediction and were not at all tied to evidence on pathophysiological causal inference. A diagnostic nosology based on symptom expression alone precludes such crucial decisions from being considered at all. The DSM-5 criterion of at least five of nine symptoms entails 256 different combinations of symptoms.^2^ What evidence could be used to distinguish between any of these combinations for consideration of intervention targets, related to the variety of ways patients would express those symptoms? The plethora of clinical manifestation patterns may suggest a mixture of populations with different etiologies and corresponding treatments. What evidence can be used to refine such knowledge over time, toward improvements in intervention targeting? The diagnostic criteria for MD lacks objective biological, molecular, imaging and other evidence that may shed light on etiology; including any environmental or social factors that may determine differences in symptom expression, based on etiology. As defined by the CET, the only factors that would result in changes in outcomes when targeted by interventions are etiological. Therefore, the answers to the above questions on the evidentiary basis for determining improvements in diagnostic criteria, for improving outcomes, can only be causal. Thus, it is unsurprising that the diagnostic nosology for a disorder of depressed mood has yielded such limited advances in outcomes for MD, over decades of research.

Most in the field believe that mental disorders, such as MD, are a result of the complex interactions among many categories of etiological factors (e.g., genetic, molecular, neuroendocrine, neurocircuitry, developmental, and social factors) producing clinically significant outcomes. As will be fully elucidated in a later section: The only symptoms or signs (comprising diagnostic criteria) that could result in clinically significant outcome improvement, are those that inform on the etiological factors with strongest effect on those outcomes; because those symptoms and signs are the clinically observable effects of those etiological factors.

### The self-perpetuation of a non-causal diagnostic nosology

DSM-5 has also set the conditions for the perpetuation of this problem into the future by the validity standard proposed for DSM-5 diagnostic criteria:

*“Until incontrovertible etiological or pathophysiological mechanisms are identified to fully validate specific disorders or disorder spectra, the most important standard for the DSM-5 disorder criteria will be their clinical utility for the assessment of clinical course and treatment response of individuals grouped by a given set of diagnostic criteria (p. 20)”* (35).

Unfortunately, it will be extremely difficult—or even impossible, other than by pure serendipity—to meet this “most important standard” without knowledge of “incontrovertible etiological or pathophysiological mechanisms.” The standards the DSM-5 proposed for validating a given diagnosis include prognostic prediction (i.e., “assessment of clinical course”) and “treatment response.” However, as we will see, *reliable, and accurate* prognostic prediction is mathematically dependent on causal knowledge for most distributions and thus is very hard to achieve without it. Therefore, the search for effective treatments—unguided by causal knowledge—is very unlikely to be successful, given the fundamental (and definitional) relationship between causal knowledge and treatment response, described previously (17, 18).

Thus, selecting standards for the appraisal of the validity of its diagnostic criteria that largely depend on causal knowledge considerably undermines the ability to reach such an “incontrovertible” standard, given the dependance of the advancement of causal knowledge on causal nosology. Such a standard makes the eventual inclusion of causal knowledge within the field’s diagnostic nomenclature practically impossible.

The formalized uncoupling of causation from psychiatric diagnostic nosology, which began in 1980, is further exacerbated by a much longer insidious practice in the field to *exclude disorders from the field’s domain of responsibility once their etiologies become established*. A notable example of this practice concerned the syndrome of mood, anxiety, and psychotic symptoms, accompanied by a host of somatic symptoms, known as “General Paralysis of the Insane” that was diagnosed in approximately one third of patients hospitalized with a mental illness in 1913: when it was discovered that the brains of these patients were infected with *Treponema pallidum*, revealing the treatable condition known as neurosyphilis (36, 37). With such an etiology discovered, this disorder was renamed and was transferred to the responsibility of the fields of neurology and infectious disease!

Another notable example concerns temporal lobe epilepsy where—prior to the discovery of their ictal and interictal etiologies—syndromes of disordered mood, thought, and personality fell within the responsibility of psychiatry (38), until again transferred to neurology. *It is reasonable to expect then that, barring a paradigm shift to causality, the field of mental health will be a perpetual store of unexplainable and non-scientific collection of patterns of symptoms with no grounding to causal mechanisms. Only with this shift will understanding of the root causes of mental disorders deepen, and the field’s scientific causal understanding will become consistent with that of other medical and health disciplines.*

### Limited progress in advancing causal knowledge and its consequences for intervention

Many in the field may believe the characterization of the limited progress in causal knowledge described in this article is unfair, and that advancements in causal knowledge on mental disorders is far greater than what has been stated. To this concern, the following should be considered: For an advancement in causal knowledge to be relevant for advancing a medical field, it would need to be translated into an intervention approach that targets the discovered cause, and the effect of the intervention would need to be observed through clinically meaningful outcome improvements (for a sufficiently large proportion of the relevant population believed to have the disorder). For which mental disorder has such advancements been observed since 1980?

Consider, again, MD (as an illustrative example of the more general problem, identified): By 1980, most of the seminal discoveries related to the catecholamine and serotonin theory of depression had already been published, and medication designed to increase the synaptic availability of norepinephrine, such as Tricyclic Antidepressants and Monoamine Oxidase Inhibitors, had already been developed, achieved FDA approval, and were routinely used in clinical practice (39–41). The Selective Serotonin Reuptake Inhibitors (SSRIs) were released a little more than a decade after DSM III, but many of the studies implicating serotonin reuptake in depression were published prior to 1980 (42, 43). And since 1980, the primary psychotropic agents employed in the treatment of depression are based on causes/theory related to catecholamines and serotonin (44). Regarding psychotherapy, Aaron Beck began to publish his theory on the cognitive etiology of depression in 1963, which remains the foundation of Cognitive Behavior Therapy (28), and Sigmund Freud published *Mourning and Melancholia*, a theory on the etiology of depression that remains foundational for the psychoanalytic treatment of depression, in 1917 (26). Since 1980, several newer theories on the etiology of depression have been proposed, including hopelessness theory (45), stress-diathesis theory (46), theories related to stressors and loss (47), theories related to neuroplasticity (48) and regulation of neurogenesis (49), theories related to HPA-axis hyperactivity (50) and impaired glucocorticoid receptor feedback inhibition (51), theories related to social signal transduction for immune system and inflammation (52), theories related to excessive secretion of macrophage monokines (53), theories related to deficits in theory of mind (54), and theories related to metabolic hibernation (55), to name only a few.

To say the least, the field has not arrived at a consensus on the causal effect of any of the factors identified in its etiological theories, and very few empirical studies have been conducted to gather evidence that can arbitrate between alternate theories. This problem of unsettled theory also plagues the field for other mental disorders (56, 57). This problem has profound implications in that the causal theory of a disorder that is accepted by a field provides the primary map the field can use to consider the interventions that it should deliver to improve patient outcomes.

It is often argued that results from clinical trials on many mental health psychosocial interventions/psychotherapies (i.e., evidence-based treatments or EBTs) provide strong empirical support for the field’s progress. However, when an EBT is implemented in the real world, the effectiveness is typically much weaker than in the trial (58–62). A critical factor that determines the effectiveness of a trial-based intervention in the real world is the fidelity of the intervention (63, 64), but high fidelity is hard to reproduce in usual care settings (63–65). Moreover, there has not been widespread adoption of EBTs (65–67). EBTs have, in general, been designed to target a small number of causes, and are delivered in a relatively uniform way to all individuals, according to their fidelity standards. However, if the etiology of a mental disorder is complex, individuals would be expected to vary by causal factors. Accordingly, if the disorder is of complex etiology, intervention strategies would need to be personalized and precise in targeting those factors to be effective for a large proportion of the population with the disorder for which the intervention is designed.

An intervention determined to be efficacious or effective in a clinical trial indicates the discovery of scientific knowledge that may—or may not—translate to the improvement of outcomes in the population, at sufficient scale and scope. In considering all mental health psychosocial interventions/psychotherapies that have been established to be efficacious or effective in a clinical trial together: To our knowledge, there is not a strong evidence base on the numbers of individuals in the population who have received any of these interventions near their fidelity standard, nor on the numbers of these individuals who have shown improvement for their mental disorder as a result of having received these interventions. Without such knowledge, it is hard to justify the conclusion that results from the field’s clinical trials provide strong evidence for the field’s progress. It is not surprising then that in a large international survey on public perception of mental health and its intervention, people receiving professional mental health intervention reported that the intervention was not very helpful for their needs (68).

Moreover, effects observed in Randomized Clinical Trials (RCTs) have been called into question by several comprehensive studies of the mental health clinical trials literature. The previously-mentioned Leichsenring et al. umbrella meta-analysis of the intervention effects of 3,782 RCTs concerning most major mental disorders concluded that the effects of most interventions for most disorders were small and that bias in reporting of results was common (5). Their findings were consistent with an earlier (2004) umbrella meta-analysis by Trikolinos et al. of 100 meta-analyses for 1,024 clinical trials of mental health interventions, examining interventions that had been studied at least five times in three different years. Their findings in general confirmed relatively small effects for interventions, with diminishing magnitudes of effect with further trials (69).

Such problems in effectiveness of treatment should be unsurprising, given the implications of the CET. Because outcomes can only be improved through intervention on their causes, and treatments have largely developed in a field with only rudimentary knowledge on causes (implicitly acknowledged by the field with the persistence of a non-causal nosology in 2013, with the release of DSM-5), then it would be extremely surprising if interventions developed to improve outcomes in the population would have yielded findings that generalized to typical care settings, especially for disorders of complex etiology (which most in the field believe is the case for most mental disorders). It has been proposed that a host of causal factors—and their complex interaction—are involved in treatment effectiveness and use (70, 71). Such factors involve aspects of the treatment itself, patient characteristics, clinician characteristics, and aspects of the service delivery setting. Most clinical trials can only assess a few of these factors and interactions. A similar generalization problem is evident in biomarker research, in that, to our knowledge, biomarker discovery has hardly ever penetrated routine clinical practice, despite decades of research (3, 72).

Bringing a causal research focus to research and practice for mental disorders—which will be described in a later section—would enable the identification of promising interventions, and the ability to discover both causal knowledge and the effectiveness of interventions through clinical research. Such a causal research focus can also enable the field to rigorously address complexity, given that the complexity of a disorder is highly related to inter-individual differences on how a disorder may be expressed through the specific causal factors controlling that expression. Thus, causal knowledge is necessary for the application of *personalized precision medicine* in complex causal systems.

A comprehensive review article on progress for MD recently documented the limited progress that has been achieved for that disorder:

“*In spite of decades of research, relatively little is known about its pathogenesis, other than that risk is largely defined by a combination of ill-defined genetic and environmental factors*… *Despite a broad armamentarium for major depression, including antidepressants, evidence-based psychotherapies, non-pharmacological somatic treatments, and a host of augmentation strategies, a sizable percentage of patients remain non-responsive or poorly responsive to available treatments*” (73).

### How causal factors for mental disorders are conventionally discovered

Conventionally, the field accepts randomized, controlled experiments as the almost exclusive vehicle for inferring causes. There are, however, three main problems with such extreme reliance on experimental research for causal inference:

1. *Human randomized controlled experiments can rarely be conducted in human etiological research* because it is usually impractical, unethical, or simply impossible to manipulate hypothesized etiologic factors in human studies.
2. *Animal randomized controlled experiments can be conducted, and provide some insight on causal factors*, but there are obvious problems with relying on animal experiments to inform etiology on mental disorders, not only because of the obvious differences in biology but also the social and environmental factors linked to mental health.
3. *Human randomized controlled experiments (the ones that can be conducted) can typically only examine one or a handful of causes at a time*, and—within the space of possible causes for mental disorders—such methods will be woefully inadequate for decoding disorders of complex etiology where multiple (thousands of clinical and behavioral; up to millions of molecular) causal factors, and the complex, multi-level, relations between them, will be necessary to understand the expression of disorder.

As such, results will typically oversimplify the complex etiology of mental disorders and miss numerous factors that cannot be studied in randomized controlled experiments. Because of these limitations, current literature pertaining to etiology is largely comprised of human observational, correlational studies that cannot reliably distinguish causal from non-causal correlates for outcomes and estimate causal effects. Although publications of such studies often include a perfunctory “correlation is not causation” cautionary statement, their findings are discussed, at least implicitly, in causal/mechanistic terms, or at least have that potential. Such findings then form the foundation of the field’s knowledge on the etiology of its disorders, as defined in its theoretical literature.

The limited employment of methods suitable to infer complex causation, and the lack of a diagnostic nosology to encode the causal knowledge that is discovered, can significantly undermine the field’s progress, even in cases of exciting serendipitous discovery. For example, lithium was first discovered to be efficacious for bipolar disorder in 1949 (74), yet the mechanism of action of lithium on bipolar disorder is still poorly understood (75–77). Although such facts are disappointing, they are also unsurprising, when methods are not used to clarify complex causation for mechanistic understanding, and a diagnostic nosology is unavailable for such understanding to be encoded. By encoding such mechanistic understanding in diagnostic criteria, research subjects can be classified by causal mechanism to contribute to future advances in causal mechanistic discovery. In a later section, we outline rigorous scientific methods that are available to advance discovery on complex etiology, and we provide an account of how a causal diagnostic system can be established to encode the causes that are discovered.

### Causality and the complexity of mental disorders

It is commonly believed that mental disorders are complex. Yet, despite such widely held belief, the methods and tools conventionally employed to study and treat mental disorders cannot possibly approach the level of complexity most in the field believe these disorders to have. The limitation of RCTs to discover complex etiology was previously described. Next, we describe the complexity constraints of non-causal diagnostic nosologies, such as DSM.

**FIGURE 1:**
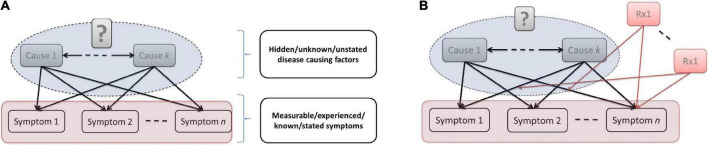
**(A)** Causal structure implied by DSM. **(B)** Application of implied causal structure for treatment.

Figure 1 illustrates how a non-causal diagnostic nosology makes the encoding of complex causal structure impossible. Figure 1A shows the only type of causal structure that would justify any DSM-5 criterion. Figure 1B then shows the intervention implications of this causal structure.

Essentially the DSM diagnostic system assumes that some unknown/unstated causal factors (inside the blue oval) *somehow* generate a range of measurable/experienced symptoms. The *core of the diagnostic classification* (inside the pink rectangle) is a set of patterns over these symptoms—without reference to what is causing them. Figure 1B shows that the clinician cannot use the nosology to manage the patient, but instead has to superimpose (by reference to external sources on therapeutics) the full set of known interventions that affect these symptoms without any means to prioritize or choose among them. As can be seen, it is impossible to use a non-causal diagnostic nosology to encode complex causal structure. In a later section, we describe how a causal nosology can be developed to provide the knowledge required to advance outcomes for mental disorders, given their complexity.

Despite the fact that the field endorses methods and diagnostic approaches that cannot address complexity, the argument is commonly made that the lesser progress for mental disorders, compared to other medical disorders, is due to their greater complexity. To argue that mental disorders are *too complex* to expect significant progress, while also establishing a nosological paradigm requiring simplistic causal structures, significantly undermines the field’s progress.

Three counterarguments demonstrate the serious error in the *mental disorders-are-too-complex-to-expect-much-progress* argument, as shown in Table 2.

**TABLE 2:** Three arguments for why mental disorders are not too complex to expect much progress.

**Argument 1: Simplicity in discovery**
What appears from ignorance to be too complex, once the actual causes are revealed, becomes simple. The historical example of Syphilis-induced psychosis is very instructive. A priori this psychosis appeared to be too complex, however a simple infection by a simple bacterium fully explains the downstream cascade of clinical manifestations and suggests a simple treatment (antibiotics), diagnostics, and preventative measures.
**Argument 2: Advances for other complex medical diseases**
In recent years diseases outside the realm of mental health, that could have been considered too complex have been decoded and great outcome progress has been achieved. Cancer research is a prime example whereby a myriad of somatic mutations can cause what was initially perceived to clinically be the same type of cancer. By embracing complex biological mechanisms and assays, the field of cancer research has revolutionized the understanding of cancer and simultaneously has advanced its therapeutic armamentarium from “kill every cell that divides” drugs to drugs that target specific molecular pathways responsible for cancers, often on an individual patient basis (7). Another example is gastric ulcer disease which was also considered to be the result of complex environmental, social, and personality interactions yet was shown for large swaths of the population to be due to a bacterium that is reasonably easy to treat (126).
**Argument 3: Complex systems “Control points”**
Complex systems have discoverable and manageable “control points” that reduce the complexity and give means for effective interventions. Consider Cardiovascular disease: whereas the lifestyle behaviors, environmental effects, genetics, molecular and cellular biology, interacting systems represent millions of variables, the essential causality needed to guide both research and effective prevention and treatments can be reduced to a simple causal axis: coronary artery damage à reduced blood flow to cardiac muscle à CAD including MI. Discovery of this “essential causality” of the disease has effectively driven highly effective preventative, diagnostic and therapeutic discoveries as noted previously.

A common accompanying argument to the *mental disorders-are-too-complex-to-expect-much-progress* argument, is based on the premise that such a vast number of causes necessarily entails that any discovered cause will have very small effect on outcomes. Accordingly—it is argued—a focus on causal discovery will not result in needed advancements for mental disorder outcomes, because interventions targeting causes with small effects will not have appreciable effect on outcomes (78–83).

We note that—if this phenomenon of a vast number of causes, with more-or-less equally small effects, were to be the case—the discouraging message about lowering expectations about the results of causal discovery would apply to the entire scientific pursuit for mental disorders, because mental disorder outcomes can only improve by targeting their causes. Moreover, such an argument also implies that the etiology of mental disorders is non-discoverable, because of the magnitudes of the sample sizes that must be employed to study hypothesized causes with such vanishingly small effects.

However, before we condemn ourselves to such defeatist conclusions, we can find hope in the notion that the reality of mental disorder etiology is likely to differ significantly from the accounts on which such conclusions are based. Causes will, likely, vary on their magnitudes of causal effect, with a small number of causes having much larger effect than the vast majority of other causes, given the causal structures that will likely be discovered to underlie mental disorders (84–88). A hierarchical account of causal structure of complex systems (in contrast to a flat structure, where a great many causes directly lead to a target variable of interest), has been well described for many complex systems in nature, and a hierarchical account of such systems has been described as an essential feature of their adaptive qualities (84–88). This implies that whereas direct (unmediated) causes may theoretically be many and with small effects, a handful of upstream indirect (mediated) causes (with strong causal effects) can regulate numerous downstream causes (with small effects). These upstream regulators can then be targeted by therapeutic interventions. In addition to the above considerations, it has been firmly established that biological and other natural systems (as well as artificial systems that have redundancy and resiliency properties) have network structures that obey power-law distributions of connectivity, and are relatively sparse, but well connected (84–88). This entails that in data related to mental disorders, the vast majority of variables have a small number of direct causes, contrary to postulates about too many direct causes with small effects.

We conclude that—ultimately—the arguments for the vast complexity of mental disorder etiology, entailing a great number of tiny effects, is—at its core—unscientific. These arguments are justified by research from within the field’s paradigm, but they cannot be falsified or verified by such research; *because this research does not employ methods able to discover large effects within complex etiology*. Arguments that causal discovery may not serve as a productive route for the field’s progress can only be verified or falsified by empirical evidence obtained using methods suitable to determine complex causation for mental disorder outcomes, and rigorously estimate the magnitudes of causal effects from the array of causes that are discovered. We detail these methods in the next section.

Like many in the field: We believe that mental disorder etiology is, likely, very complex. We believe the complexity of this etiology is eminently discoverable, using methods that will be introduced in the next section; producing advances for other medical disorders of complex etiology such as cancer (89–93). Accordingly, we look to a paradigm that would not place the complex etiology of mental disorders beyond the reach of science. Rather, we envision a scientific paradigm suitable to achieving advances, based on whatever level of complexity mental disorder etiology is discovered to have. Such a paradigm will: be infused with rigorous methods that can elucidate causal structures at high levels of complexity; determine the magnitudes of effects for those factors discovered to be contained within the causal structure of mental disorders; and target those causes with the largest magnitudes of effect with interventions in clinical trials, to advance knowledge on interventions that can improve outcomes. Further, such a paradigm will make scientific discoveries that can directly support improved outcomes for people with mental disorders. Such access is only possible if those patients are diagnostically classified in such a way that would reveal that their outcomes can improve by interventions guided by causal knowledge. Accordingly, a scientific paradigm anchored by a causal diagnostic system is the only way to make a field’s science available to improve outcomes for patients at risk for those outcomes.

Next, we detail the paradigm we envision, that is causally based and empirically directed. We recommend the steps that can be taken to bring a mental disorder science to benefit the hundreds of millions of individuals now burdened with such disorders.

## Toward a new scientific paradigm on mental disorders: Discovering the causes of mental disorder outcomes, and encoding such discoveries in diagnostic nosology

Fields responsible for the prevention and treatment of mental disorders cannot advance by continuing to apply the same paradigm that has produced, over decades of research, the inadequate results that we have described. The major structural barriers we have identified concern: (1) the employment of scientific methods unsuited to gain causal knowledge on mental disorders, and (2) the employment of a diagnostic nosology unsuited to classify patients by cause. Changing the paradigm will entail enormous challenges, but expectations of different results with application of the same methods is unlikely to bear positive results in advancing outcomes for mental disorders.

Notwithstanding this pessimistic appraisal of the field’s ability to advance with its current paradigm, there are considerable opportunities available to enable significant advancements to occur, and a practical route by which such a change in paradigm may be realized. Despite the significant and complex challenges that will have to be addressed for such change to occur, the direction forward involves a commitment to two broad paradigm changes: (1) the discovery of causal knowledge must become the overarching goal for research on mental disorders, and (2) the encoding of causal knowledge must become the overarching goal for any diagnostic system on mental disorders. In this section we chart the road to this change in paradigm.

### The discovery of causal knowledge must become the overarching goal for research on mental disorders

We cannot continue to depend on human observational correlational studies or human etiological experiments for the knowledge needed to improve mental disorder outcomes, and animal experiments can only provide preliminary knowledge, which must be confirmed in rigorous human etiological research. Fortunately, such rigorous human etiological research is made possible through significant breakthroughs in the fields of Causal Data Science (CDS), including several causal discovery algorithms from observational data and powerful mathematical theories specifying conditions under which non-experimental data can be used by specific algorithms to reliably infer underlying causal processes. These causal discovery algorithms can uncover causal relationships, even with very high levels of complexity, large numbers of factors, and unmeasured confounders, and provide estimation of quantitative causal effects (17–22). Such quantitative estimates can identify promising intervention targets (from knowledge of the level of change in outcome that would be estimated to result from targeting a causal factor with intervention) (17–22). These methods, in addition to having rigorous mathematical proofs in their support, have strong empirical track records for detecting causes using validation data sets where causes are previously known. Parallel innovations in predictive modeling allow for accurate diagnostic and prognostic models.

Next, we provide a brief non-technical overview of CDS methods and algorithms, followed by a section that reviews the application of these methods to medical disorders, including mental disorders. Interested readers may benefit from reviewing the two textbooks that are widely used in the CDS field (17, 18), and the articles that will be referenced in the section on the application of these methods, to medical and mental disorders.

#### An overview of causal data science methods

Causal Data Science methods solve problems that fall into two broad categories: *causal structure discovery* and *causal effect estimation*, and enable the examination of multiple causal factors from the same study:

*Causal structure discovery* identifies those factors that are causal of specifically defined outcomes, and the relations between the various causes for those outcomes. These include: direct (i.e., unmediated) causal relations among direct and indirect (i.e., mediated) causes of outcomes, and the factors participating in the chains of cause →outcome (i.e., terminating at a defined outcome); Such causal knowledge is typically represented as a causal graph (network). Models of causal structure for a specific outcome variable, discovered by causal structure methods, allow an investigator to answer the question: Does factor A cause outcome B? (e.g., Does a lack of social support cause depression?). They also describe, reveal and explain non-causal correlations that are generated by confounding structures among the correlated variables.

*Causal effect estimation* determines the magnitude of change of outcome per unit of change (due to intervention) on the causal factors. Such causal effect estimation methods enable an investigator to answer the question: If I were to intervene on causal factor A (identified in the causal structure model), what is the amount of change I would expect to observe in outcome B? (e.g., if I intervened to increase the amount of social support by 50%, what is the amount of improvement in depression that I would expect to observe, or what is the reduction in probability of depression that I would expect to observe)? The same applies to concurrent manipulations of multiple causal factors. These causal effects are represented as conditional probability distributions of each variable, given its direct causes. Together with the causal graph, they comprise a complete causal model from which the full joint distribution as well as marginal and conditional distributions among the modeled variables can be estimated.

Causal mechanisms among a set of variables with a specific causal structure generate data that have corresponding statistical properties. By imposing mild restrictions on the distributions we study, a correspondence between the dependencies and independencies in the data, and the causal structure generating the data, can be established. This correspondence is the pillar on which causal discovery algorithms are based: Specifically, the common distribution restrictions are known as the Causal Markov Condition (CMC), and the Faithfulness Condition (FC). The CMC requires that a variable is statistically independent of all other variables, except its (direct and indirect) effects, given its direct causes (17, 18). The CMC entails a number of behaviors that are commonly accepted in macroscopic causality (i.e., excluding quantum mechanics and other phenomena not relevant to the macroscopic world where health sciences operate). Such explained behaviors are, for example: that confounders create spurious correlations that can be controlled, if we control the confounders; or in causal chains (A→…X→. .→Z) all variables are correlated, however, if we condition on any intermediate variable, the variable upstream and downstream of it will cease to be correlated; etc. The FC posits that independencies in the data are only due to the CMC, and holds in the vast majority of distributions.

The CMC, together with the FC, enable algorithms to infer the causal structure (including the presence of unmeasured variables), given statistical independence relationships estimated from observational data. Thus, the CMC—with the FC—are the keys for inferring causal relationships vs. associational relationships, and for estimating causal effects.

The variety of structure discovery algorithms generally fall in two categories. The *constraint-based methods* implement the CMC + FC by strategically conducting conditional independence tests to discover and orient causal edges. The *score-based methods*, on the other hand, calculate posterior probability (or other) scores of causal models and identify models with causal structures that maximize the scores given the data. Causal structure discovery methods discussed here have been proven correct under broad assumptions (CMC, FC, and causal sufficiency), while more recent algorithms also tolerate certain violations of those assumptions (17, 18).

Due to the complexity of the causal discovery problem, computational efficiency and scalability represent the major challenges for causal discovery algorithms. Even in systems with a moderate number of variables (e.g., more than a few dozen variables), it is computationally inefficient and can be impossible to examine all conditional independence relationships, because the number of all possible conditional independence tests grows exponentially with the number of variables. Therefore, causal structure discovery methods implement systematic search strategies to ensure the statistical properties in the data are examined iteratively in a computationally efficiently manner so that the discovered causal structure is correct. Some algorithm variants are scalable to millions of variables even with modest computing equipment and without sacrificing correctness. The efficiency of these methods enables determination of causal structure with such large numbers of variables in practical time-frames for investigators. By comparison, it is entirely impossible for humans to conduct the vast number of calculations needed to correctly infer causality and is also impossible to conduct randomized experiments that infer complex causal relationships (because that would require manipulating up to all variables simultaneously in the system under study).

Causal structure discovery algorithms can determine complex causal structures, comprised of all chains of direct and indirect causes—and their points of interaction—terminating in variables measuring mental health outcomes. Within such causal chains, distinctions between more proximal (e.g., stimuli/triggers for immediate psychopathological responses) and more distal (e.g., genetic propensities to respond to specific stimuli/triggers) causal processes can be defined.

**FIGURE 2:**
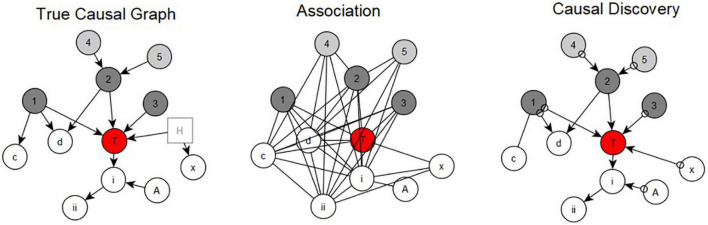
Causal data science vs. association methods for determining causal structure.

Figure 2 illustrates the use of causal discovery methods to discover causal structure, as compared to relying on associations. The left panel shows the true local causal structure around a mental disorder target outcome, T, with its direct (unmediated) causes shown in dark gray and its indirect (mediated) causes shown in light gray. Variables non-causal of T are in white, and are all statistically associated with T (univariately or multivariately), because they are effects of T, or are effects of a cause of T.

According to the Common Cause Principle (a special case of, and following from, the CMC + FC) proposed in 1956, any factor that is an effect of a cause of another variable will be observed to correlate with that variable, confounded by their common cause (94). Thus, C will correlate with T because it is an effect of 1, d will correlate with T because it is an effect of 1 and 2, and x will correlate with T because it is an effect of H (an unmeasured or “hidden” variable). Moreover, any effects of c, d, x, i and ii will also correlate with T (and any of their effects, and so on), by the Common Cause Principle. Also: A will correlate with T, conditioned on i (i.e., in a multivariate model that has variable i as covariate). Thus, mental disorder outcomes of complex etiology, should be expected to generate much larger proportions of statistical associations that are non-causal than those that are causal. The most pernicious effect of confounding relates to causes unmeasured in a given study. The danger of hidden confounding is shown by hidden variable H, resulting in x’s statistical association with T.

Thus, applying association-based (middle panel) methods that are conventionally used to identify promising intervention targets or mechanisms of mental disorder outcomes (e.g., univariate associations, poorly constructed regression models and structural equation diagrams, coefficients of predictor models, relevance and network science models, principal component analysis factor loadings, etc.) will identify associations among all variables (other than A) with T. Therefore, using evidence from statistical association to identify a cause that might indicate an intervention target is fraught with errors. Relying on the strength of association to determine causation is also not a good strategy because, given the true causal structure, joint distributions exist in which non-causal variables (such as d, i and ii, x) have larger statistical associations with T than do any individual true causes of T. However, applying proper causal discovery algorithms can resolve up to all causal relationships, depending on which factors have been measured and what is the underlying causal structure (compare the right panel to the left). Note that using causal algorithms that do not assume causal sufficiency (i.e., that all common causes of measured variables are measured) can lead to identification of potential hidden confounders. However, in other cases, statistical relationships in the data can exclude the possibility of hidden variables (e.g., between 2 and T). Detailed description of theory and algorithms for identifying hidden confounders can be found here (18).

Determination of causal structures of mental disorder outcomes is necessary for identifying which factors are causal—or non-causal—of outcomes. However, such knowledge is ultimately in the service of answering a singular question, the answer to which the field’s progress depends: *What factors will produce clinically significant improvements in outcomes, were they to be targeted with interventions?* The threshold levels of improvements that would be regarded as clinically significant can be determined by domain experts for particular mental disorder outcomes and taking into account patient preferences. The magnitudes of estimated change, produced by targeting a particular cause contained in a valid model of causal structure, can be determined by methods of CDS, which would help domain experts to identify those most promising for clinically significant change.

Causal effect estimation determines quantitative causal knowledge, for example, how much improvement in a mental disorder outcome, such as suicidal behavior, would be expected if a certain causal factor, such as family conflict or amygdala activity (identified in a causal model), were lessened with interventions. The Do-Calculus method (17) enables the estimation of causal effects. It conducts effect estimation by selecting the appropriate covariates to condition based on the causal model, previously determined with causal structure discovery methods described above (or leveraging known causal structure). The Do-Calculus method is guaranteed to produce unbiased effect estimation given the correct causal structure (17). Do-Calculus combined with qualitative causal structure discovery algorithms is critically different from conventional methods such as structural equation modeling (95), path analysis (96), matching (97), and propensity scoring (98). Because the conventional methods of the structural equation family are generally hypothesis-driven and examine only a small fraction of possible causal structures governing the data, they are likely to miss the true causal structure and result in biased estimates of causal effects. Moreover, even without any hidden variables present, the number of possible models is astronomical for even a few dozen variables, making specification of the correct model via SEMs, matching, or propensity scoring, millions of times less likely than winning the lottery (even with a modest number of variables)! These methods essentially presuppose we have solved the qualitative structure causal discovery problem before hitting the modeling stage. Causal structure discovery together with causal effect estimation methods, using Do-Calculus, circumvent these shortcomings.

A crucial advantage of the application of causal structural discovery with Do-Calculus estimates is the capacity to efficiently determine those few causal factors that have potential to change outcomes when targeted with interventions and exclude the great many correlates without potential to change outcomes with interventions, thus preventing dead-ends in intervention development efforts before such efforts are launched. The effort to develop new interventions, and then to test their efficacy and effectiveness in clinical trials, is a very long and expensive process. A 2016 economic study of the research and development of 106 randomly selected medications from 10 different pharmaceutical companies estimated the cost before achieving FDA approval was 2.6 billion dollars, each (99). There is great interest in the field to define methods that can identify promising intervention targets from data collected in observational research so that clinical trials can be focused on only those most promising targets (100). CDS methods can substantially improve the field’s capacity to do this.

This discussion has focused on using CDS methods to exclude confounded relationships between factors on outcomes, in the identification of intervention targets. As described, because of the extensive connectivity of causal biological systems, numerous patterns and clusters of symptoms that form DSM-5 diagnostic criteria for particular disorders will be observed. However, without knowledge of the causal structure, findings from these clusters of associated factors cannot convey the information needed about which interventions will improve outcomes versus interventions that are useless.

#### Track record of causal data science methods for determining causes with observational data

Although the CDS methods described in the previous section are unconventional for etiological research on mental disorders, the correctness of algorithms for learning causal relationships and estimating causal effects from non-experimental observational data has been established firmly via several decades worth of experiments in machine learning and econometrics (17–22). The works of Judea Pearl (Turing Award recipient for his causal discovery work), Sir Clive Granger (Nobel Prize recipient in Economics for so-called “Granger Causality” methods), and of Spirtes, Glymour and Scheines, in particular, stand out in their impact and broad acceptance in CDS. Most recently, the awarding of the 2021 Nobel Prize in Economics to David Card, Joshua Angrist, and Guido Imbens for their work applying causal inference methods to real world observational data, leveraging “natural experiments,” demonstrates the importance of applying methods for observational data to be able to obtain causal knowledge (101). We refer readers to the numerous mathematical proofs of correctness for a variety of algorithms in Pearl and Spirtes et al. (17, 18).

CDS methods have yielded important advances in medical fields such as cancer, diabetes, rheumatic disease, and infectious diseases (102–110). Since 2016, a series of published studies have demonstrated their applicability to mental disorders with data sets of modest size, containing variables typically measured in longitudinal risk factor research studies on mental disorder outcomes. Table 3 summarizes the findings of some of these studies.

**TABLE 3:** Track record applying causal data science to mental disorder outcomes.

Study description	Sample	Etiological factors discovered for intervention targeting
Longitudinal study of hospitalized injured children for causal factors within complex system of psychopathology (e.g., anxiety, depression, externalizing symptoms) 1 year after hospital discharge (127)	174 hospitalized, injured children	Hospital pain, acute stress; low heart beat-to-beat variability in hospital; Stress 3 months after discharge; externalizing symptoms 3 months after discharge. FKBP5 gene, COMT gene SNPs.
Longitudinal study to predict post-traumatic stress and identify causal factors in hospitalized injured children, 1 year after discharge (128)	174 hospitalized, injured children	Acute stress; acute pain; resting heart rate; parent’s acute stress; child’s harm avoidance shortly after discharge. COMT, CRHR1 gene SNPs.
Longitudinal study of PTSD trajectories 5-months after discharge from emergency department (129)	250 adults seen in ER following injury	Negative cognitive appraisal, distress in the ER; high urinary norepinephrine; avoidance 1 week and 1 month discharge.
Longitudinal study (LONGSCAN data set) to determine causal factors for post-traumatic stress and negative self-image at age 8 (130)	1,374 children identified in infancy at risk for maltreatment	Peer acceptance; low “physical competence” at age six; feeling physically threatened or unsafe; post-traumatic symptoms, for negative self-image.
Longitudinal study to determine to determine causal factors for social and occupational functioning in Schizophrenia (131)	276 adults diagnosed with schizophrenia	Socioemotional functioning, motivation
Studies to determine the direction of causal relationships between categories of post-traumatic, substance abuse, and internalizing symptoms, in a veteran cohort and two civilian cohorts (132)	240 veterans and two civilian cohorts (*n* = 79 and *n* = 116)	PTSD is causal of depression and not vice versa.
Longitudinal study of police academy recruits to determine causal factors for PTSD and Depression 1 year after start of police duty (133)	207 police academy recruits	Work adjustment, social adjustment, startle reactions to low threat stimulus, peritraumatic distress. MR and HDC gene SNPs.
Longitudinal study of adults hospitalized for an injury in Australia to determine causal factors for PTSD, 1 year after discharge (134)	586 injured adults	Pre-injury social anxiety and alcohol abuse; ruminative thoughts on why injury occurred; anxiety, depression, numbing, avoidance 1 month after discharge
Study to use causal factors to determine treatment response for adolescents with depression using data from the treatment of adolescents with depression study (TADS) (135)	282 adolescents	Psychosomatic symptoms, school missed, view of self, treatment expectations, attention problems determined the patients’ response to specific treatments.
Longitudinal study of the causal determinants of drinking behavior (136)	362 adults with Alcohol Use Disorder	Social anxiety, perceived stress
Longitudinal study of the causal determinants of drinking behavior (137)	926 adults, 22% Alcohol Use Disorder	Agreeableness/social support, negative affect, low conscientiousness/attention, externalizing symptoms. Prefrontal cortex. Frontoparietal networks.

In summary: A mature scientific literature has developed demonstrating the utility of CDS methods for identifying causal factors with potential as intervention targets, and a developing literature has emerged since 2016 indicating that these methods can be successfully applied to identify causal factors of mental disorders. As the conventional methods applied to learn the complex etiology of mental disorders for decades have not produced the advances needed, we recommend the application of these methods to contribute to the imperative to discover robust knowledge on the complex etiology of mental disorders.

### The encoding of causal knowledge must become the overarching goal for any diagnostic system on mental disorders

We cannot continue to depend on a non-causal diagnostic system to contribute to improved mechanistic understandings or advances in prevention and treatment of mental disorders. Progress depends on the capacity to encode causal knowledge in diagnostic nosology. Determining the path to establish a causal diagnostic nosology may seem daunting to a medical field that has practiced under a non-causal one for over 40 years. In this section, we recommend the steps necessary to help get the field back on track.

To begin, we must recognize the central role of causal reasoning in clinical practice, in all branches of medicine. A patient presents to a clinician with a problem—the clinician gathers information about the patient’s presenting problem—to determine: *What-is-the-matter-with-the-patient?* If the clinician determines that the answer to this question would lead to future compromise of the patient’s health or functioning (at any future timescale, even minutes or seconds), the clinician seeks to deliver an intervention to prevent such compromise. Thus, at the core of clinical practice, lies the search to answer the question: *What-is-the-matter-with-the-patient?* This question is answered through causal inference on the clinical data (i.e., symptoms, signs, including laboratory results), collected about the patient. The central question to be addressed in this section is: *How can clinical data about a patient be used to determine the patient’s risk for clinically significant compromise to their health and functioning; and*—*if the patient is determined to be at such risk*—*how can the clinical data also inform on the interventions to use to diminish the risk?* As we will discuss: The answer to these questions require causal inference on the clinical data, to determine *what-is-the-matter-with-the-patient*; and the most powerful causal inferential tool a medical field can offer its clinicians, is its diagnostic nosology. In this regard, it is worth considering the meaning of the answer, *“You have Major Depression”*; to a patient who asks, *What-is-the-matter-with-me?*

Effective clinical practice requires clear causal thinking, which requires a precise language to support it. A medical field’s causal vocabulary is its diagnostic nosology, which supports causal thinking on each occasion it is employed; because on each of these occasions, the clinician understands that the name used for the patient’s disorder indicates a cause (i.e., *what-is-the-matter*) of a future clinically significant outcome (i.e., a compromise to the patient’s health or functioning).

Language powerfully influences thought (111). As we will show, a non-causal diagnostic nosology diminishes the capacity to apply causal thinking to help patients. We also suspect that the 40-year history of conducting its science and practice without a causal vocabulary has diminished the field’s capacity to even notice that causal knowledge is missing. Perhaps the strongest evidence for this conclusion is contained in the fact that four significant revisions to the field’s non-causal diagnostic system have been released since 1980, without any serious consideration to integrate causal knowledge in diagnostic criteria (or the absence of significant alarm across the field on the occasion of each revision, because the decision to continue with the non-causal nosology was justified by a lack of progress on causal knowledge).

In this section, we lay the foundation for returning to a causal diagnostic system for mental disorders. We start by offering an account of first principles of the diagnostic process in medicine, using a precise causal language, consistent with the language introduced in Section “The discovery of causal knowledge must become the overarching goal for research on mental disorders.” Then, we offer three steps necessary to allow a causal diagnostic system to be established, such that progress on improving outcomes for mental disorders can be achieved. As we will show, a causal diagnostic nosology is the only means that a medical field possesses for enabling the fruit of its science to be available to improve patient outcomes.

#### First causal principles on diagnosis in medicine

To take first steps toward a causal diagnostic nosology, it is important to consider the central role of causality in the diagnostic process in medicine (112–115). Such consideration requires key concepts to be kept distinct. A diagnostic nosology that is able to encode knowledge that can improve outcomes will be based on distinctions between:

1. *Symptoms and Signs*, including laboratory test results (i.e., what constitutes the clinical data forming diagnostic criteria);
2. *Pathologies of the Human Body* (i.e., what is “disordered” in a medical disorder); and,
3. *Clinical Outcomes* (i.e., the objective of patient care: to improve patient prognosis by intervening to reduce the probability of future compromise to the patient’s health or functional capacity).

In essence, the knowledge necessary to improve a medical disorder is based on how each of these three categories relate to each other causally. Disorders that are defined by a non-causal nosology cannot keep these categories distinct-enough to consider how causality relates them in the service of improving patient outcomes. Knowledge on the causal relations among variables within these distinct conceptual categories allows for diagnosis to inform patient prognosis, and the means to change prognosis with intervention.

Diagnostic criteria are formed from symptoms and signs (including laboratory test results) expressed by patients, because these symptoms and signs are *observable to clinicians*. Symptoms and signs are the clinical data for diagnostic criteria, because they inform on *pathological processes* that are not directly subject to observation. A symptom is the patient’s subjective expression of pathology (e.g., pain, anxiety). A sign (including laboratory test results) is the objective expression of pathology (e.g., patellar reflex response following reflex hammer percussion, blood pressure reading, blood glucose test result, amygdala activity on Functional Magnetic Resonance Imaging, etc.). Thus, as clinically observable effects of pathology, symptoms and signs are the material available to clinicians to infer the pathology that made them available for observation. The quality of any diagnostic criteria for informing about such underlying pathologies must (at some level) be based on the performance of the symptoms and signs of the criterion-set, to accurately classify patients with the pathology.

**FIGURE 3:**
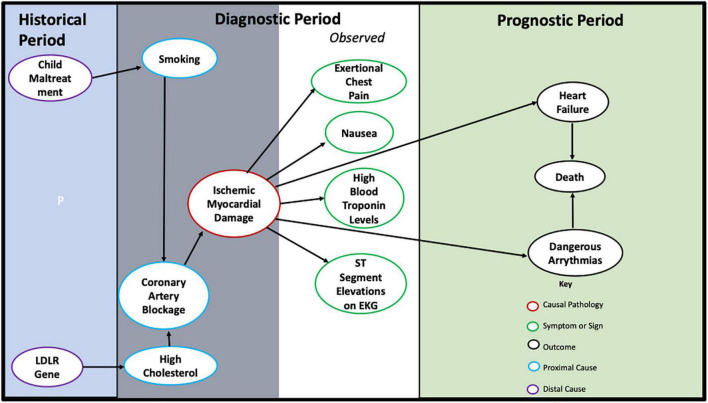
A causal model for the diagnosis of myocardial infarction.

In Figure 3, we illustrate these processes in a model of the diagnosis of Myocardial Infarction (MI). The model is centered by the time period when a medical diagnosis is relevant to patient care (i.e., the Diagnostic Period, in Figure 3). In this period, a patient presents to a clinician with a presenting problem (e.g., chest pain). The clinician conducts an assessment to gather information to determine the relevance of the patient’s presenting problem for the patient’s future prognosis (at any future timescale, even minutes or seconds). The future prognosis defines the Prognostic Period in Figure 3. In the Diagnostic Period, information on symptoms and signs (including any lab test results that are ordered) are collected to determine whether the patient meets diagnostic criteria for MI (or for any other condition, with prognostic implications).^3^

Assessment information on causal factors contributing to the pathology are also collected in the Diagnostic Period. In Figure 3, we distinguish between proximal causal factors, and distal factors. We define proximal causal factors as those factors exerting effect on the pathological process during the Diagnostic Period. Thus, a proximal cause is a potential intervention target. A distal cause has either already exerted its causal effect on the pathology, or on a proximal cause, prior to the Diagnostic Period; or reflects an enduring characteristic of the patient, such as sex, race, culture, or genes. Such distal causal factors may help for diagnostic classification or the understanding of disorder mechanism, but they cannot represent targets for intervention at the time of diagnosis. Distal causes are placed in the Historical Period in Figure 3. Distal causes can be helpful for preventative interventions prior to the Diagnostic Period, because these causes are causal of pathology and proximal causes.

As can be seen in Figure 3: Symptoms and signs of exertional chest pain and nausea, and signs of ST segment elevation on EKG and blood troponin levels, are effects of the pathology of ischemic myocardial damage, and are the clinically observable material used to diagnostically classify patients with this causal pathology. Such a diagnostic label thus encodes the pathology of ischemic myocardial damage, through its established diagnostic criteria.

The ischemia that leads to the heart damage is caused by the proximal cause of blockage of the coronary arteries. This proximal cause is an effect of the other proximal causes of smoking, and high cholesterol levels, shown in Figure 3. The model in Figure 3 also shows several distal causes in the Historical Period: Childhood maltreatment and the Low Density Lipoprotein Receptor (LDLR) gene. Although these don’t typically represent intervention targets, they can be used to diagnostically classify patients, to understand mechanism, and to indicate possible pre-clinical prevention strategies (e.g., prevention of child abuse, use of genetic information for early screening).

The effects of ischemic myocardial damage on congestive heart failure, dangerous arrythmias, and death, define the clinically significant outcomes of the Prognostic Period. Although evidence of blocked coronary arteries, high cholesterol, or smoking, are not part of the diagnostic criteria of MI, the collection of this information can be very important for intervention targeting, because these proximal causes are causal of the pathology, causal of outcomes. As illustrated in the causal model in Figure 3, the diagnosis of MI entails straightforward approaches to intervention that can improve prognosis, including medical or surgical interventions to increase cardiac blood flow, smoking cessation, medications to lower cholesterol levels, antiarrhythmic drugs, and drugs to increase contractility of the heart.

In summary: Causality is a quality of clinical knowledge that connects diagnosis with prognosis (and, especially, the means to favorably change prognosis). Symptoms and signs forming diagnostic criteria are the clinically observable effects of pathology. Outcomes that are considered by a medical field as clinically significant—defining the patient’s prognosis—are also the effects of the pathology. The diagnostic name assigned to a patient’s clinical condition, classified by diagnostic criteria (formed from those symptoms and signs), simultaneously informs on patient prognosis and on the means to change prognosis with intervention.

#### The application of first causal diagnostic principles to mental disorders

Perhaps the single greatest innovation in medicine, over its history, is the establishment of diagnostic nosological systems based on etiology (116). All branches of medicine, including mental health until 1980, organize their diagnostic nosological system by etiology, in order to leverage this innovation for the field’s progress. When a diagnostic name communicates etiology, it makes available a field’s scientific literature to help patients; otherwise, such literature is inaccessible. This is the case because:

*To know whether any scientific finding may be relevant to improve a particular patient’s prognosis, the patient must be classified to belong to a particular category of patients, who a medical field believes would respond to particular interventions (at any future timeframe from classification, even minutes or seconds).*

Of course, patients can only be classified in this way if the field’s diagnostic criteria have been constructed from those symptoms and signs (including diagnostic test results) commonly expressed by patients within diagnostic categories, because those symptoms and signs are the clinically observable effects of pathologies, causal of prognosis, for those patients. By defining diagnostic categories in such a way, over its history, all branches of medicine powerfully make their medical science available to patients receiving their care.

Establishing a causal diagnostic nosology for mental disorders would seek to apply the first causal principles on medical diagnoses, reviewed in the previous section, to define mental disorder diagnostic criteria. Success in this task is greatly facilitated by the existence of a widely accepted definition of a mental disorder. This definition forms an ideal starting point for the application of these first causal principles. Remarkably, the definition of a mental disorder offered in the DSM-5 is entirely consistent with these principles. DSM-5 mental disorders are defined as *clinically significant disturbances in an individual’s cognition, emotional dysregulation, or behavior (i.e., symptoms)*—*reflecting underlying dysfunctions (i.e., pathologies)*—*resulting in distress or functional impairmen*t (i.e., *prognostic outcomes*) (35).

Of course, the only way to use such symptoms to change resulting distress or functional impairments is by categorizing them according to their status as effects of underlying pathological dysfunctions. Any set of symptoms or signs that would be the clinically observable disturbances in *cognition, emotional dysregulation, or behavior*, would form the causal diagnostic criteria for a mental disorder, to the degree that that diagnostic criterion-set informs on pathological processes (i.e., underlying dysfunctions), causal of clinically significant outcomes.

**FIGURE 4:**
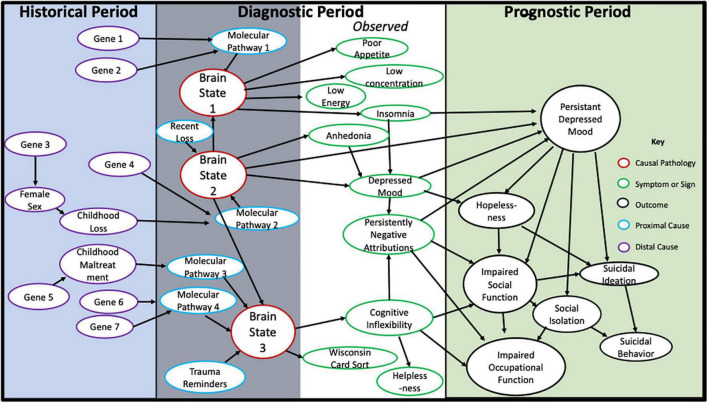
A causal model for the diagnosis of a disorder of depressed mood.

How may we use the DSM-5 definition of a mental disorder, described above, to define mental disorder diagnostic criteria, causally? We answer this crucial question by illustration with a hypothetical causal model of a mental disorder, capturing information on its natural course, similar to the account we offered on MI in Figure 3. Figure 4 provides a hypothetical model of a disorder of depressed mood.

A causal model, capturing the clinical course of a medical disorder, starts with one or more outcomes that are believed to be clinically significant. Then the causal model, based on empirical evidence, provides an account of the causeeffect relations that produce those outcomes over time. In Section “The discovery of causal knowledge must become the overarching goal for research on mental disorders,” we introduced structural causal models to describe how models, such as those shown in Figures 3, 4, can be derived. Figure 4 is hypothetical, with certain categories of variables left open to provide an illustration of how such structural causal models can be used to determine diagnostic criteria for a mental disorder. In a later section, we will detail why the field’s consensus over outcomes of greatest clinical significance will be an essential step toward establishing a causal diagnostic nosology.

Imagine that the causal model shown in Figure 4 was obtained from a research study applying CDS methods to an existing longitudinal data set. The aim of this study was to determine a causal model that could reveal diagnostic criteria for a disorder of depressed mood. The investigators sought a data set that would contain information related to current DSM-5 symptoms of MD, later outcomes of functional impairment, a variety of possible pathological variables (collected around the time of data collection on symptoms), and a variety of other proximal and distal biopsychosocial variables that could plausibly affect outcomes through pathology. The investigators used the same definition for clinically relevant timeframes used in the presentation of Figure 3 for MI (e.g., the Diagnostic Period), and the same definition of variable categories (e.g., Proximal Causes).

The data set chosen was determined to be suitable for the investigators’ goals because it included: (1) A data collection wave containing variables that could be symptoms or signs, biological variables that could be pathologies or biological proximal causes, and environmental variables that could be environmental proximal causes (data from this wave was defined as the Diagnostic Period); (2) A data collection wave—6 months after the Diagnostic Period wave—containing variables that could be clinically significant outcomes, including functional impairment categories and suicidal thoughts and behaviors (data from this wave was defined as the Prognostic Period); and (3) Data available from before the Diagnostic Period wave or about enduring characteristics of subjects (e.g., demographic characteristics, genes) that could be distal causes (variables from this category were placed in the Historical Period). This data set was analyzed with the causal structure discovery methods described in Section “The discovery of causal knowledge must become the overarching goal for research on mental disorders.” The only information that was included for data processing by the algorithms implementing these methods—apart from data on each variable for each subject—was the time period of the variable, for time ordering of variables by category (i.e., Historical, Diagnostic, Prognostic Periods) to result in the structural causal model shown in Figure 4. Recall that this study is hypothetical, with certain variable categories left open, to illustrate key points.

As can be seen in the Figure 4, a model that sought to examine causes and effects of impaired social and occupational functioning related to depressed mood found cascading downstream causal effects in the Prognostic Period. These prognostic effects (of pathological causes or their clinically observable effects), cascade from the variable of persistent depressed mood, to symptoms of hopelessness, social isolation, and suicidal ideation and behavior.

These prognostic outcomes are either the direct effects of three (unnamed) Brain States in the Diagnostic Period, or clinically observable effects of these Brain States (mediating Brain State effect on outcomes in the Prognostic Period). The Brain States may be considered pathologies for the outcomes in the Prognostic Period, similar to how Ischemic Myocardial Damage was considered the pathology for the diagnosis of MI in Figure 3. For mental disorders, it is an empirical question: where the causal pathology of the prognostic outcomes of greatest clinical significance will be located in the human body. Although we suspect they will be found in specific variants in brain function (e.g., activity level of a brain region, an aspect of brain circuity or functional connectivity), broadly defined in Figure 4 as *Brain States*, the specific pathology within brain functioning, or other bodily functions, will emerge with empirical causal research. In a later section, we will offer a general definition of mental disorder pathology to, at least, guide future research in the crucial pursuit of pathological processes, causal of outcomes.

As can be seen, the three pathological Brain States effect different clinically observable symptoms and signs, for different outcomes of prognosis. Brain State 1 is causal of four neurovegetative symptoms (and only insomnia is causal of prognosis, through persistent depressed mood); Brain State 2 is causal of anhedonia, which is causal of depressed mood (which is causal of persistent depressed mood and hopelessness in the Prognostic Period); and Brain State 3 is causal of cognitive inflexibility (which is prognostically causal of social and occupational dysfunction). The joint effects of Brain States 2 and 3 lead to specific effects for prognosis. In particular, the effects of depressed mood and cognitive inflexibility directly cause a persistent negative attribution bias leading to interpreting events in the world negatively. This bias perpetuates the depressed mood, and affects social and occupational dysfunction, directly.

Each Brain State is directly influenced by proximal causes: Brain State 1 by molecular pathway 1; Brain State 2 by molecular pathways 2 and 3, and recent interpersonal loss; and Brain State 3 by molecular pathway 4, and exposure to trauma reminders. In turn, these Brain States, and their proximal causes, are differentially influenced by distal causes: specific genes, demographic factors, and social variables in the Historical Period. As the causal pathologies (e.g., Brain States) and their proximal causes occur in the Diagnostic Period, they can represent important intervention targets (to the degree that the causal research methods estimated that they had large enough quantitative effect on outcomes, and interventions are available to target them). As described previously, distal causes do not represent intervention targets, but can provide important information to determine disorder mechanism, contribute to accurate patient classification by causal pathology (e.g., history of child maltreatment, and genetic tests for genes 5, 6, and 7, can be helpful for classifying patients for a disorder or sub disorder related to Brain State 3), or to point to the means to prevent pathology, for patients encountered earlier in the course of illness.

Additionally:

1. Since Brain State 2 is causal of Brain State 1 and Brain State 3, and the outcome of persistent depressed mood, all the effects of Brain State 1, Brain State 2, and Brain State 3 will be observed to statistically correlate with this outcome (as common effects—with the outcome—of Brain State 2).
2. All effects of each Brain State will be observed to statistically intercorrelate (as common effects of the respective brain state). Evidence for such intercorrelations inform on prognosis and intervention targeting, by informing on respective pathological brain states.
3. The clinically observable symptoms or signs in the Diagnostic Period can be divided into two categories: (i) causal of a prognostic outcome via mediation of the effect of causal pathology (e.g., insomnia), or, (ii) non-causal of a prognostic outcome (e.g., poor appetite, low energy). A symptom or sign in the former category can represent an intervention target, whereas a symptom or sign in the latter category can only inform on causal pathology.
4. Many of the symptoms currently comprising criteria for MD are included in Figure 4. However, the utility of the symptoms for guiding patient care through diagnostic criteria entirely depends on knowledge of the pathologies that caused their availability for clinical observation. As described previously, the DSM criterion of at least 5 of 9 symptoms for a diagnosis of MD yields 256 possible combinations. The relevance of any of these combinations for patient care can only be understood through knowledge of causal pathology.
5. The measurement of the causal variables (e.g., how each brain state or molecular pathway is measured) is left out of the figure for clarity of illustration of key concepts. Measurement is a complex conceptual and technical issue in causal modeling that goes beyond the scope of the article. In essence, since—strictly speaking—causes are hidden (i.e., causal modeling concerns inferences on the process generating the data), all data is an effect of a cause. A medical field manages this problem by its prior knowledge on the measurement of variables that it believes would closely correspond to distributions from causes).

Regarding the use of Figure 4 to construct a diagnostic criteria for a mental disorder: There are many subtleties to such decisions, but for illustrative purposes we propose the following: The close causal interconnections between downstream outcomes, starting from the variable of persistent depressed mood—mapped to a small set of pathologies—would argue for a singular disorder, of three subtypes (defined by the three respective Brain State pathologies). Diagnostic criteria for the disorder, and its subtypes, are defined by clinically observable symptoms and signs, and evidence on proximal causes and distal causes: Depression Subtype 1 by Brain State 1, neurovegetative symptoms, molecular pathway 1, and genes 1 and 2; Depression Sub-type 2 by Brain State 2, anhedonia and depressed mood, recent loss, history of childhood loss, molecular pathway 2, genes 3 and 4, and female sex; and Depression Sub-type 3 by Brain State 3, cognitive inflexibility, Wisconsin Card Sort results, helplessness, molecular pathway 3 and 4, traumatic reminders, a history of child maltreatment, and genes 5, 6, and 7. This diagnosis, and its subtypes, straightforwardly point to intervention targets: the respective Brain States, their proximal causes, and their clinically observable effects, causal of outcomes (to the degree they have large estimated causal effect, and intervention is available for targeting).

Thus, well established causal diagnostic principles can greatly enhance the ability to improve patient outcomes for mental disorders, provided that causal knowledge advances so that it can be encoded in diagnostic nosology. In contrast to the difficulty in the use of DSM-5 diagnoses for enabling decisions for intervention targeting (discussed in Section “Causality and the complexity of mental disorders” for Figure 1), our discussion of the diagnosis for a disorder of depressed mood, reviewed in this section for Figure 4, reveals the clarity of the translation of diagnostic names for intervention targets that can be achieved with a causal diagnostic nosology for mental disorders. In Section “The discovery of causal knowledge must become the overarching goal for research on mental disorders,” we described methods available to enable the discovery of this knowledge for such encoding.

Based on our review of causal concepts, and their application to clinical diagnosis and clinical care, we recommend three steps for the field to take, for launching processes that can lead to the establishment of a causal diagnostic nosology for mental disorders.

#### Three steps to establish a causal diagnostic nosology for mental disorders

##### Step 1: Establish scientific and clinical consensus on the outcomes of greatest clinical significance that would result from disorders of cognition, emotional regulation, and behavior

The effort to establish a causal diagnostic system can easily be undermined by efforts to maintain the same diagnostic divisions that have, essentially, guided the field for decades. Efforts to maintain these divisions would be reflected in research questions that are constrained by existing conceptions of mental disorders, such as: *What are the causal pathological processes for obsessive compulsive disorder? What are the causal pathological processes for bipolar disorder?* We must leave our preconceived notions of what we will find, and go where the data may take us. We suspect the process of establishing a causal diagnostic system will entail considerable revisions of diagnostic categories.

Constraining causal research questions to existing diagnostic categories undermines the establishment of causal diagnostic nosology, because the causes that must be discovered are the pathologies effecting the outcomes a field believes are of greatest clinical significance. We may start to look for the field’s consensus on its most clinically significant outcomes from within the pages of DSM-5; but in so doing, we are confronted with a problem. Depending on how one counts, we may (with most conservative standards) find 157 distinct disorders of cognition, emotional regulation, and behavior in DSM-5 (within its disorder categories and subcategories). Do these 157 represent the field’s consensus on its outcomes of greatest clinical significance? If not: Where in DSM may we find how this number may be reduced? Determining outcomes of greatest clinical significance is of crucial clinical importance, involving priorities for clinical care (i.e., intervening to prevent outcomes of greatest clinical significance).

We believe a particularly difficult barrier to arriving at such a consensus concerns the conflation of the concept, *diagnosis*, with the concept, *outcome*, that is commonly observed in the field. Often terms for these concepts are used interchangeably in reference to mental disorders. When nosology excludes causal meaning, distinctions between these concepts can lose their relevance. Not only will this conflation preclude the field from arriving at a definition of the outcomes of greatest clinical significance to use to anchor a new causal diagnostic system, it will also obscure the ability to consider an intervention target as anything other than a symptom included within the diagnostic criteria.

Within the current mental health paradigm, a patient is diagnosed with a disorder because a specific criterion-set of symptoms is observed. Then, the goal of intervention is to change the state of the patient, so they no longer meet this diagnostic criteria (or have a reduction of symptoms, from the criterion set). This phenomenon is also reflected in the similarity between the entry criterion commonly used in a mental health clinical trial (i.e., the patient meets diagnostic criteria) and the definition of intervention success, used in that trial (i.e., the patient no longer meets diagnostic criteria). On one level, it is easy to understand how this use of diagnostic criteria, for outcome definition, can be justified: *Symptoms forming diagnostic criteria can persist into the future, and can be associated with high levels of distress and functional impairment.* However, when these terms are used interchangeably, without awareness of what ties them together, the capacity to employ intervention to change outcomes, and the capacity to encode intervention targets within diagnostic nosology, is considerably undermined.

When a DSM-5 diagnosis is made, the reason it may also be considered as an outcome is with the assumption that it will persist into the future. Such persistence can only occur if the symptoms comprising diagnostic criteria were the effects of underlying, untreated causes. Thus, no matter the level of distress or functional impairment that may accompany any symptom expression, the only systematic means of stopping its persistence, is via the targeting of their causes with intervention. This is the reason why right lower quadrant abdominal pain, with rebound tenderness on abdominal examination, are considered as symptoms and signs for the diagnosis of acute appendicitis, rather than as intervention targets to reduce their own persistence into the future, as outcomes. If considered as intervention targets and outcomes, the patient’s pain and abdominal tenderness could simply be targeted with analgesics, and (depending on the dose), this intervention could be observed as effective for treating these outcomes.

The goals for the setting of diagnostic criteria and for determining outcomes of greatest clinical significance are quite different. The former goal concerns the accurate classification of patients, whereas the latter goal concerns the setting of clinical priorities toward outcomes that are most consequential for health or functional capacity. Symptoms forming DSM-5 diagnostic criteria may vary widely in their levels of clinical significance. For example, the DSM-5 diagnostic criteria for a Manic Episode includes seven possible symptoms. Two are described, as follows: (1) *Symptoms of excessive talking and pressure to keep talking*, and (2) *Excessive involvement in activities that have a high potential for painful consequences (e.g., engaging in unrestrained buying sprees, sexual indiscretions, or foolish business investments)*. The latter symptom would usually represent a much greater clinical priority than the former, given its potential prognostic consequences.

Decisions on the criterion-set of symptoms and signs for mental disorders must follow the consensus within the field on the most important prognostic outcomes it is responsible to prevent. The DSM-5 definition of mental disorders has already set a reasonable starting point to begin to consider a manageable set of outcomes of greatest clinical significance: *Levels of distress and functional impairment*. As illustrated in the discussion of Figure 4, causal modeling methods can be used to determine biological variants (i.e., pathologies) causal of the most significant levels of future distress or psychosocial functional impairments (including its subcategories of work and relational function and independent living). Such modeling would reveal those symptoms of *cognition, emotional dysregulation, or behavior* that may be most causal of the most clinically significant levels of distress and functional impairment. Those symptoms that are not causal of distress or functional impairment have utility for diagnosis via their role as clinically observable effects of pathology.

Although we have proposed levels of distress and functional impairment as reasonable starting points for the field’s consensus on outcomes of greatest clinical significance, such decisions are of crucial importance for launching a causal diagnostic system for mental disorders, and require the field’s consensus on these matters.

##### Step 2: Define mental disorders, by the pathological causes of those outcomes of greatest clinical significance, and set diagnostic criteria by the clinically observable effects of those pathologies

In considering the 157 distinct mental disorders contained in the DSM 5, we ask: Is this number too high? Is it too low? What evidence should be used to determine this number? We can answer these questions practically: The number of distinct mental disorders should be based on the unique value of the information, conveyed by a distinct diagnostic name, for patient prognosis and the means to change prognosis with intervention. Diagnostic categories with highly overlapping prognoses, and similar intervention approaches, should be merged. Diagnostic categories with distinct prognostic trajectories, and correspondingly distinct intervention approaches, should be kept, or added. Of course, from the previous discussion, distinctions between diagnostic categories (and subcategories) for mental disorders must be based on causal pathology to inform prognosis, and the means to change it.

Distinct mental disorders would be defined based on the mappings of distinct sets of causal pathologies, with distinct sets of outcomes. Such mappings would be used to determine definitions of mental disorders, and their subcategories. For example: The same (small) set of causal pathologies, mapping to many distinct sets of outcomes, provides evidence for a small number of mental disorders with diverse effect on outcomes. This would be a fortunate result because then interventions directed at a small number of causes can have great impact. Alternatively, many distinct sets of causal pathologies mapping to those same number of outcomes, would define many distinct mental disorders, requiring different intervention strategies, and defined with distinct diagnostic criteria.

Once distinct disorders are defined in this way, subcategories of disorder can be determined, similarly. If a set of interrelated causal pathologies are found to map to a specific set of outcomes, specific pathologies within the set or specific antecedent causes of the pathology may reveal district opportunities for intervention. Such evidence would argue for specific disorder subcategories. In the hypothetical example for a disorder of depressed mood shown in Figure 4, diagnostic subcategories were defined by brain state. Depending on the decisions on distinct disorders, and their subcategories, defined in this way; decisions on the diagnostic criteria to use, follow straightforwardly. The diagnostic criteria for a disorder (and its subcategories) is formed from the symptoms and signs that are the effects of those causes, optimized toward the classification of patients by cause.

It is hard to anticipate where the symptoms and signs, that currently define DSM-5 disorders, will land when this approach is followed. We recommend that the field remain open to considerable reorganization of its diagnostic categories, through learning from the data in this way.

Next, we provide more detail on two categories of variables, important for consideration in defining mental disorders.

##### Defining the pathology of mental disorders

The central goal of diagnosis is to inform on pathology, causal of clinically significant outcomes. Diagnostic criteria enable this type of inference because they are formed from the clinically observable effects of that pathology. Defining such pathology for a mental disorder is difficult, at present; because the field’s causal knowledge is not advanced, and the field has not encoded causality in nosology for over 40 years. Accordingly, to launch a new causal diagnostic system, we expect there will be vigorous and healthy debate on the definition of mental disorder pathology. However, the bounds of this debate may be constrained by the nature of pathology, its relationship to prognosis, and guided by empirical evidence.

*Mental Disorder pathologies must represent variants on biological processes, causal of clinically significant outcomes. Thus, any consensus on a clinically significant outcome (e.g., functional impairment, suicidal behavior, violent behavior) can serve to constrain the definition of mental disorder pathology; because the pathology would*—*in some way*—*concern a biological variant causal of whichever outcome was defined to be clinically significant.*

Such a discovery of biological variants, causal of clinically significant outcomes, would constrain the space of possibilities for definitions of mental disorder pathology, by the use of causal evidence. The use of such empirical evidence to define mental disorder pathology then, constrains the space of possibilities for mental disorder diagnostic criteria. Such criteria is reached by the field’s consensus—based on its causal literature—on the clinically observable effects of the pathology that would best classify patients for that pathology.

The human biological system is, obviously, highly complex with a great many processes interacting with each other, to determine outcomes. What would define a biological variant as pathological, and another as contributing to the pathology? Although there are obvious subtleties in this distinction that await further discussion and debate in the field, we would define as pathological those biological processes most informative of clinically significant outcomes. Usually, these would be the biological variants most directly causal of outcomes, and which may mediate the effects of other biological (and non-biological) factors on the outcomes (e.g., Myocardial Ischemic Damage in Figure 3; and Brain States 1, 2, and 3, in Figure 4).

It is hard to anticipate exactly which biological processes will prove pathological vs. antecedent for mental disorders, without a more mature causal literature. We suspect the pathology will be found in brain circuity, causal of outcomes, and symptoms of cognition, emotion, and behavior. Antecedent molecular pathways, coded by upstream genetic variants, will offer exciting opportunities for intervention targeting, by their contribution to pathology in brain circuitry.

##### Determining the role of the social environment for producing clinically significant outcomes

The social environment will likely have a strong causal role in determining clinically significant outcomes for mental disorders. It is important, however, to carefully consider its specific role in producing these outcomes. Outcomes for mental disorders—defined by systems producing cognition, emotional dysregulation, and behavior—are caused by pathological systems of the human body. These pathologies have causes, including from the social environment (e.g., reminders of a traumatic event, loss of an interpersonal relationship, stressors at work). Thus, the effect of any social environmental factor on the outcome is mediated by a variant in bodily function. Social environmental factors can inform important targets for intervention, and can be used to define subcategories of disorder, but their effect on the outcome is mediated by causal pathology. Distal social environmental causes (e.g., history of trauma, history of loss) can be very helpful for understanding mechanism, for diagnostic classification, and prevention of pathology.

##### Step 3: Align causal discovery, diagnostic criteria, intervention development, and clinical trials, so that traction can be achieved in outcome results over time

The establishment of a causal diagnostic nosology can be seen as an optimization problem that will advance over time by the use of causal evidence. Such advancements will occur—as they have for other medical fields—when the processes of diagnostic classification, causal discovery, intervention development, and clinical trials, are aligned. The problem requiring optimization is to determine the best set of the symptoms and signs to use, to classify individuals by pathology, causal of clinically significant outcomes. The set of symptoms or signs with poor classification performance will place too many individuals without the pathology, within the diagnostic category; or miss too many individuals with the pathology, so they are classified as not belonging to the diagnostic category. Such optimization is, of course, dependent on causal evidence for the selection of those clinically observable, pathological effects to use to optimally classify individuals.

In turn, optimizing diagnostic criteria advances causal discovery, by better classifying research subjects by causal pathology. Of course, confidence that a sample of subjects, classified by causal diagnostic criteria, possess the pathology defining their diagnostic category, enhances causal discovery research to refine causal knowledge. Such reciprocal advances support intervention development and clinical trials, by yielding knowledge to classify individuals who would be expected to respond to interventions targeting those pathologies (or their proximal causes). Clinical trials of those interventions, for those individuals so classified; provide generalizable knowledge on the interventions to use, for those diagnosed in the population.

Such evidence also informs the quality of the diagnostic criteria and the causal knowledge used in the clinical trial. Evidence of improved patient outcomes in the clinical trial, that follow interventions targeting discovered causes, by patients classified by cause; generates confidence in diagnostic classification and causal discovery. Evidence of lack of such effect, stimulates questions about the quality of the criteria used for classification, and the accuracy of causal discovery, or both. These alignments have led to the transformations of many medical fields, for the improvement of patient outcomes. Thus, any diagnostic system that might be proposed for mental disorders—DSM-6, Research Domain Criterion (RDoC), or The Hierarchical Taxonomy of Psychopathology (HiTop) (117–120)- could only lead to needed advances when aligned with scientific discovery and clinical trials, as described.

Arguments that a causal diagnostic nosology must await “incontrovertible evidence” on causes must be treated with great caution, because they miss the central role of causal discovery for enabling progress in diagnostic classification. The strength of causal evidence—like every form of scientific evidence—will advance gradually over many studies until becoming “incontrovertible.”

## Conclusion

The result of 40 years of progress for improving mental health outcomes was reviewed and noted to be insufficient to the needs of patients and comparatively lacking the progress of most other health science fields. Viewing this problem with a causal lens, we have presented evidence that the overarching barrier limiting the achievement of these results is the non-causal paradigm that has guided mental health research and practice for decades. We cannot imagine that results will be any different in the next 40 years without a significant change in paradigm, because *outcomes can be improved through intervention on their causes, and in no other way*.

This article has described an approach to paradigm change that involves two broad and integrated commitments to change: *a commitment to the application of methods suitable to infer causes at scale and speed*—*given the complexity of mental disorders*—*and a commitment to encode the results of this causal discovery within the diagnostic criteria for mental disorders.*

Although such changes in paradigm will certainly entail challenges, there are reasons to believe that such a magnitude of change is feasible and can lead to success, assuming the field is able to mobilize in the directions we advocate. First, major advances in new types of omics assays, neuroimaging, electronic health records and digital health produce enormous amounts of information to drive discovery. Second, major advances in causal and predictive data science can readily be leveraged for discovery from the enormity of the information that is now available. Third, the workforce is now being trained in the generating and capturing of this information, and in the computational methods available to leverage it. Universities are now releasing increasing numbers of scientists and technologists with the understanding and proficiency needed to apply the methods and approaches we have identified as being necessary for change. These individuals should be vigorously recruited to join the great effort needed, working along with previous generations of mental health researchers and practitioners, to enact the transition to a causal science and practice of mental disorders.

## Author contributions

GS and CA: conceptualization, project administration, and supervision. GS, LB, CA, and SM: investigation and writing—review and editing. GS: funding acquisition and writing—original draft. All authors contributed to the article and approved the submitted version.

## Footnotes

1
*Granted, there might be haphazard luck/serendipity in targeting causal factors.*

2 We thank one of the reviewers of this article for pointing out this fact about the DSM-5 diagnostic criterion for Major Depression.

3 A medical field will try (usually, implicitly), to set the time-bounds of diagnostic periods, to when—in the natural course of illness—clinical information would be available for common clinical presentations, that are informative on clinically significant outcomes, within a time window sufficient to change these outcomes with intervention.
